# Long-Duration Sound-Induced Facilitation Changes Population Activity in the Inferior Colliculus

**DOI:** 10.3389/fnsys.2022.920642

**Published:** 2022-07-07

**Authors:** Alice L. Burghard, Christopher M. Lee, Emily M. Fabrizio-Stover, Douglas L. Oliver

**Affiliations:** Department of Neuroscience, University of Connecticut School of Medicine, UConn Health, Farmington, CT, United States

**Keywords:** plasticity, sound-evoked activity, spontaneous activity, electrophysiology, mouse, auditory system

## Abstract

The inferior colliculus (IC) is at the midpoint of the auditory system and integrates virtually all information ascending from the auditory brainstem, organizes it, and transmits the results to the auditory forebrain. Its abundant, excitatory local connections are crucial for this task. This study describes a long duration sound (LDS)-induced potentiation in the IC that changes both subsequent tone-evoked responses and spontaneous activity. Afterdischarges, changes of spontaneous spiking following an LDS, were seen previously in single neurons. Here, we used multi-channel probes to record activity before and after a single, tetanic sound and describe the changes in a population of IC neurons. Following a 60 s narrowband-noise stimulation, a subset of recording channels (∼16%) showed afterdischarges. A facilitated response spike rate to tone pips following an LDS was also observed in ∼16% of channels. Both channels with an afterdischarge and channels with facilitated tone responses had higher firing rates in response to LDS, and the magnitude of the afterdischarges increased with increased responses to the LDS. This is the first study examining the effect of LDS stimulation on tone-evoked responses. This observed facilitation *in vivo* has similarities to post-tetanic potentiation *in vitro* as both manner of induction (strong stimulation for several seconds) as well as time-course of the facilitation (second to minute range) are comparable. Channels with and without facilitation appear to be intermixed and distributed widely in the central nucleus of IC, and this suggests a heretofore unknown property of some IC neurons or their circuits. Consequently, this sound-evoked facilitation may enhance the sound-evoked output of these neurons, while, simultaneously, most other IC neurons have reduced or unchanged output in response to the same stimulus.

## Introduction

While responses of individual neurons to sounds vary between and within auditory structures, the canonical response of an auditory neuron to suprathreshold stimuli is an elevation of firing rate shortly after the stimulus onset and a return of firing rate to baseline shortly after stimulus offset (Chen, 1998; Wang et al., 2005). Some neurons may exhibit an increased firing rate evoked by sound offsets, but these responses generally decay within milliseconds. However, there are neurons in the auditory system with prolonged responses after the offset of a sound in the inferior colliculus (IC) (Ono et al., 2016).

The IC is an essential relay center for the ascending auditory information traveling to the forebrain, and integrates input from brainstem and descending cortical connections (Cant and Oliver, 2018). The neuronal composition of the IC is heterogenous, and neurons can be categorized into subtypes based on their spiking response to depolarizing input: regular, adapting, pauser/buildup, onset, and bursting (Peruzzi et al., 2000; Sivaramakrishnan and Oliver, 2001; Tan and Borst, 2007; Tan et al., 2007). Recently, a study of well-isolated, single neurons in the central nucleus of the inferior colliculus (ICC) revealed a subpopulation in which firing rates remain above baseline for seconds to minutes after the stimulus offset (Ono et al., 2016). This long-duration, sound-evoked afterdischarge (LSA) required a long-duration sound (LDS) stimulus at least 20 dB above threshold and lasting at least 20 s, and it was observed in ∼20% of studied neurons. The afterdischarge response is in contrast to the canonical auditory response because the response can persist for minutes after the sound offset.

What are the consequences of exposure to a LDS? One might expect neural adaptation, unlike the LSA response. One example of this may be the suppression of ongoing neural activity due to prolonged exposure to a sound that seems to correspond to the phenomenon of residual inhibition of tinnitus (Feldmann, 1971; Roberts et al., 2008; Galazyuk et al., 2017). On the other hand, with LSA, the neural firing beyond the offset of a sound is an increase in neural activity above baseline and suggests a change lasting for seconds if not minutes. In brain slice preparations, neurons in the IC can exhibit long-term potentiation (LTP) in response to tetanic stimulation of their synaptic inputs (Wu et al., 2002). Such stimulation is similar to what IC neurons may experience *in vivo* during an exposure to a LDS. However, the consequences of LSA on the responses to sound are unknown.

Here, we examined how afterdischarges evoked by LDSs modify both spontaneous activity and the response to sound in the neurons of the IC. While LSA responses are observable in individual ICC neurons, it is important to understand how the LDS stimulation affects the population of IC neurons at-large since it is the population response that is presented to the next station in the auditory pathway (Ito and Oliver, 2012). The local circuits that connect different IC neuron types may produce different responses to the same sound stimulus. To investigate IC population activity, we used 16 and 32 channel probes that spanned the tonotopic map of IC so that we could present a single acoustic stimulus and record the simultaneous responses of many neurons in the anesthetized mouse. Spontaneous activity was recorded in the IC during silent periods before and after LDS presentation. We found that activity increased significantly in 16% of channels following the LDS, matching the LSA behavior previously observed in single IC neurons (Ono et al., 2016). To address whether the same LDS also induced changes in sound-evoked activity, we recorded responses to trains of tone pips before and after the LDS and found that 16% of channels had facilitated responses while nearly half of channels were suppressed. Furthermore, among the channels with facilitated responses, the sound-evoked component of the response was rarely facilitated, suggesting that the facilitated responses were often mediated by increases of ongoing spontaneous spiking, or an amplification of spontaneous and evoked spike rates. These experiments provide new insights on how LDSs may alter circuit-level activity in the ICC and its output to the auditory forebrain.

## Materials and Methods

### Animals

The experiments were performed using adult CBA/CaJ mice of either sex. This mouse strain (The Jackson Laboratory, strain: 000654) is known to retain normal hearing up to an old age (Zheng et al., 1999). All mice were purchased by JAX (The Jackson Laboratory, Bar Habor, ME) at an age of 4–8 weeks and then housed at a 12 h light/dark cycle in cages with additional nesting material as enrichment until experiments were performed. All experiments were performed in accordance with the institutional guidelines and the NIH Guide for the Care and Use of Laboratory Animals and were approved by the Animal Care and Use Committee at the University of Connecticut Health Center.

We recorded changes in spontaneous activity before and after LDS presentation in 18 animals (nine female and nine male). We additionally recorded changes of tone-pip evoked activity before and after the LDS in 6 of the 18 animals (three female and three male).

### Anesthesia and Surgery

For all electrophysiological recordings, anesthesia was induced via an intramuscular or intraperitoneal injection of ketamine/xylazine/acepromazine (90, 9, 2.4 mg/kg, respectively). Anesthesia was maintained with isoflurane in oxygen. Oxygen was provided throughout the experiments via a nose cone at a flow rate of 0.5 L/min. Depth of anesthesia was assessed via toe pinch reflex testing (∼ every 30 min) and continuous measurement of heart rate and O_2_ saturation level via a pulse oximeter (Mouse Ox, Starr Life Science Corp, Oakmont, PA).

After induction, the animals received a single dose of 0.03 mL lidocaine hydrochloride (1%), injected under the scalp at the incision site dorsal to the midbrain. The animals were then mounted on a gas anesthesia head holder (David Kopf Instruments, Tujunga, CA, United States) and received artificial tear ointment on the eyes. The body temperature was maintained at 36–38°C with a heating pad coupled to a rectal thermometer, and 0.3 mL warm saline was injected subcutaneously at 30 min intervals.

After the mouse had been placed in the stereotaxic frame and the head fixed into position with mandibular bars (pitched forward 5° from the horizontal stereotaxic plane), an incision was made above the midline. After retracting skin and muscle, a craniotomy above the right inferior colliculus (IC) was performed (covering an area of ∼3 mm lateral to the midline and about 1.5 mm caudal to lambda). A stainless-steel screw (#0–80) was then inserted into the skull over the left cortex and served as reference electrode, while a needle electrode was placed subcutaneously in the neck of the animal to serve as ground. Following this, the dura covering the IC was removed.

### Electrophysiology

All electrophysiological recordings were performed on anesthetized animals in a sound attenuated chamber (IAC, Bronx, NY, United States). Signals were collected with silicon probes (one or two shank, 16 or 32 channel linear array, length: 3 mm, 16 channels/shank, NeuroNexus, Ann Arbor, MI, United States). The electrode sites were spaced 100 μm apart. Most recordings were made with custom, two shank, 32-channel probes where the shanks with 16 channels each were 0.4 mm apart. Euclidean distances were calculated between all pairs of channels with LSA or potentiated responses in a penetration. The impedance of the electrode sites ranged from 0.22 to 1.68 MΩ. The probe was inserted into the IC (∼1–1.5 mm lateral, ∼0.3–1 mm posterior to lambda) using a manipulator (Scientifica, East Sussex, United Kingdom). The probe was advanced into the IC at an angle of 10° pitched caudal from vertical since this allowed simultaneous recording from different frequency laminae in the central nucleus of the IC (ICC). Electrode position was verified in a subset of animals via histology. For this the electrode was coated with DiI and the position in the ICC confirmed on Nissl-stained coronal sections. Electrode signals were digitized at 25 kHz with a PZ5 amplifier and delivered to a RZ5 processor (TDT, Tucker Davis Technology, Alachua, FL, United States). Storage of the signals was done via Synapse (TDT) which in turn was controlled by custom MATLAB code.

### Acoustic Stimuli

All acoustic stimuli were generated with an RZ6 auditory processor (TDT) at a sampling rate of 200 kHz. Parameters of the acoustic stimuli were defined and digitally copied using user interface software “Synapse” and MATLAB function “SynapseLive” (TDT). Sounds were presented to the animals via a free field speaker (Revelator R2904/7000-05 Tweeter, ScanSpeak, Videbæk, Denmark) at a distance of 11.5 cm from the head of the mouse at the front at an angle of 45° elevation to the animal’s head. Sound levels at the position of the mouse head were calibrated within 5 dB from 3 to 70 kHz with a 1/4 inch microphone (Precision Condenser Microphone, #377C01, PCB Piezotronics, Inc., Depew, NY, United States).

Broadband noise bursts (3–50 kHz, 75 dB SPL, 100 ms duration, 2 Hz presentation rate) were played during electrode placement. Placement of electrodes near sound-responsive neurons in the ICC was verified with sustained firing during the sound presentation. With good electrode placement, the noise bursts typically evoked large amplitude spikes on the majority of channels. Spikes thresholds were at ∼5 × noise standard deviation, and were further manually adjusted for each channel to best capture broadband noise responses while avoiding thresholding the channel noise (Supplementary Figure 1). Due to the impedance of the electrodes, we assume that most of the channels recorded action potentials from multiple neurons. Next, the frequency response area for each channel was obtained by presenting a sequence of pure tones (200 ms duration, 4–64 kHz, 0–90 dB SPL, 10 dB and 0.25 octave steps). Each tone/sound level combination was presented five times.

The main experiments investigated the effects of exposure to a LDS on spontaneous and sound-evoked activity in IC neurons. The LDS was a 1/3 octave, band-pass noise of 60 s duration, generated by applying biquad filters (12 dB/octave roll-off) to Gaussian noise. We presented up to three LDS of different center frequencies (range of 4–48 kHz) for each subject. For each LDS center frequency, the LDS was presented 30 dB above the lowest pure tone threshold across all channels at the respective frequency (minimum absolute LDS level: 60 dB SPL, maximum 90 dB SPL; mean: 73.9 dB SPL, ±1.5 dB SPL SEM). The LDS duration and intensity were chosen to maximize the likelihood of generating LSA, based on results from Ono et al. (2016). We conducted two experiments to study changes of spontaneous and sound-evoked activity before and after the LDS. In the first experiment, we measured spontaneous activity in a 30–60 s silent period before the LDS and again after the LDS for a 240 s silent period. In the second experiment, we examined tone evoked activity preceding and following the LDS by playing 3 ms tone pips (1 ms raised cosine ramps, ∼21 Hz presentation rate). The tone pips were presented in 5 s trains separated by 5 s silent gaps. The frequency of the tone pips was matched to the LDS center frequency and the level of the tone pips were either the same as the LDS level or 10 dB less. All LDS trials (both paradigms, spontaneous and tone-evoked) were presented at intervals of 10 min or longer, to allow for recovery from possible short-term plasticity induced by LDS stimuli.

### Analysis

#### Frequency Tuning

A frequency response area (FRA) was computed for each channel by presenting a pure tone sequence described in the previous section. To determine thresholds, the spontaneous rate was measured from a 200 ms window before each stimulus onset, and the response rate was measured from a 100 ms window following the stimulus onset. A 95% confidence interval was calculated from the spontaneous rate based on a Poisson distribution, by evaluating the MATLAB function “poissinv” with a mean at the average number of spontaneous spikes during the 200 ms windows. Each stimulus condition was classified as evoking or not evoking a response if the response rate exceeded the spontaneous upper confidence interval. The absolute threshold and characteristic frequency (CF) were defined by the level and frequency of stimulus of the lowest level able to evoke a response.

Because we recorded multiple channels spanning the ICC, we were able to compare changes of spontaneous and sound-evoked activity among channels with CFs varying around the LDS center frequency. We categorized channels based on whether the CF fell within (LDS = CF), above (LDS < CF), or below (LDS > CF) a 1/3 octave band around the LDS center frequency.

#### Spontaneous Activity

Spike rates before (PRE) and after (POST) the LDS presentation were estimated by binning spikes into 2.5 s bins. Spiking activity during the LDS presentation was estimated in 500 ms bins. Activity in channels were only analyzed when there was a significant response driven by the LDS. The response to the LDS was tested by comparing the spike rate of the first bin during the LDS presentation to the 95% confidence interval of the baseline spontaneous rate, based on a Poisson distribution of spike rates. The baseline spontaneous rate was measured during the silent 30–60 s PRE phase.

**FIGURE 1:**
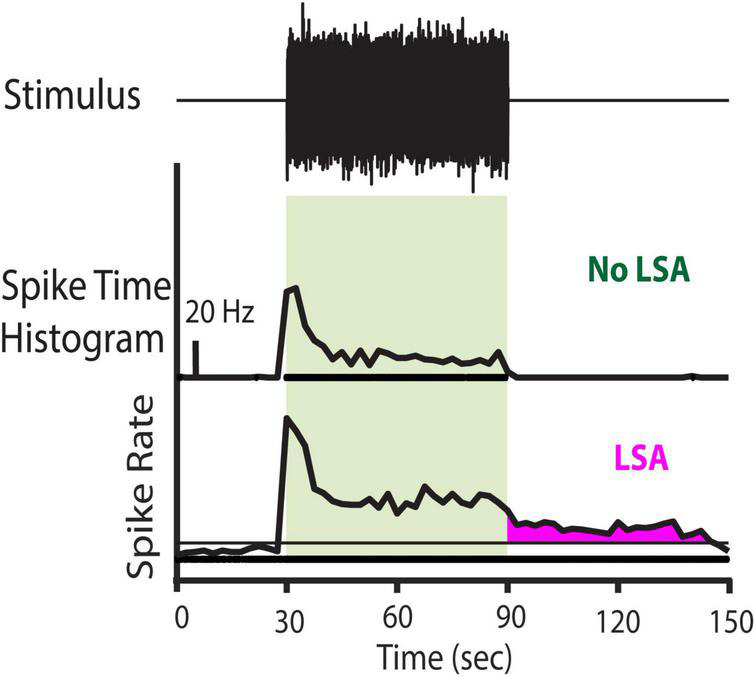
Stimulus paradigm for spontaneous activity trials and example of the presence or absence of LSA activity. **(Top row)** Stimulus Paradigm. One-third octave, narrow band noise preceded by a silent period of 30–60 s and followed by another 240 s of silence. Presence of the noise stimulus is indicated by the light green box in the other rows. **(Middle Row)** Representative Channel with no LSA activity. **(Bottom Row)** Representative channel with LSA activity. Horizontal line indicates the 95% confidence level (CL) of the spontaneous firing rate preceding the stimulus. The LSA activity is marked in magenta. To be considered a valid LSA activity, the firing rate after the LDS offset had to exceed the 95% CL of the spontaneous firing rate for at least three consecutive bins and start within the first 30 s after stimulus offset. Spike rate is depicted in Hz, the scale bar in the middle panel indicates 20 Hz.

The spontaneous firing rate POST was compared to the spontaneous PRE firing (Figure 1). To distinguish a long-duration sound-evoked afterdischarge (LSA) from noise fluctuations of spontaneous activity, POST spiking activity was considered LSA only if the afterdischarge rate on a recording channel exceeded the 95% confidence interval of the spontaneous spiking rate (measured during PRE), for three consecutive bins. Therefore, random variation of the spontaneous rate under a Poisson distribution is very unlikely (*p* < 0.001) to meet this criterion. Furthermore, the onset of the period of significantly elevated rates had to begin within 30 s of the offset of the LDS. This additional criterion excluded responses with periods of elevated firing starting well after the LDS, and therefore less likely to be linked to the LDS.

#### Tone Evoked Activity

**FIGURE 2:**
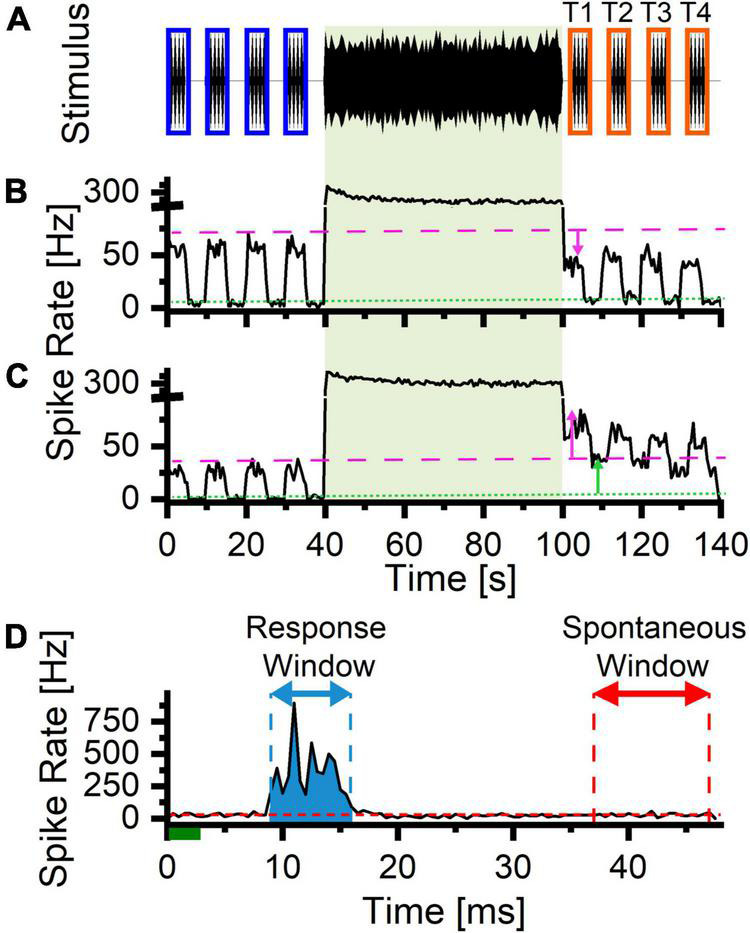
Stimulus Paradigm for tone evoked trials. **(A)** Stimulus paradigm: Tone pips (3 ms, 1 ms rise/fall time, 21 Hz, pure tones matching the center frequency of the 1/3 octave LDS stimulus) before and after the LDS. The tone pip presentation was split into four 5 s trains separated by matching 5 s silent periods. The blue rectangles represent the PRE analysis windows. The orange windows represent the matching POST analysis windows (T1–T4). **(B)** Representative example of a channel with suppression after the LDS presentation (downward magenta arrow indicates a downward shift in the tone evoked firing). The green dashed line indicates the spontaneous firing rate (during silent periods) preceding the LDS presentation (baseline_*spontaneous*_). The magenta dotted line indicates the maximum tone pip evoked response preceding the LDS presentation (baseline_*evoked*_). **(C)** Representative example of a channel with potentiation of both tone evoked and spontaneous activity following the LDS presentation (upward arrows, magenta and green, respectively). **(D)** Example a PSTH illustrating the measurement of the evoked activity (blue shaded area) and spontaneous portions (red dashed line) of the cycle. The green line represents the presentation of the 3 ms tone pip stimulus. The blue arrow indicates the response analysis window (bins above threshold rate = (95% × spontaneous rate) + (5% × peak rate), the red arrow indicates the window used to measure spontaneous activity (37–47 ms post stimulus onset). The red dashed line represents the average spontaneous firing rate. The blue shaded area represents the response area (integrated area of the PSTH of the response area – the expected average spike rate).

In the second experiment, 3 ms tone pips were played before and after an LDS presentation (Figure 2A) and the changes in the sound-evoked activity were examined. Channels were analyzed only if they had sound-driven responses to either the LDS or to the tone pips during either the PRE period or the POST period. To determine sound-driven activity to the LDS, the baseline spontaneous rate (Figures 2B,C, green dotted line) was measured from a silent 4 s period preceding the LDS. To determine whether activity was driven by probe tones, we analyzed the power spectrum of the spike histogram (0.5 ms bins) during the PRE and POST tone pip trains (spectra were computed with function “pspectrum” from MATLAB 2019a Signal Processing Toolbox). Responses to tone pips were considered to be sound driven if the four largest peaks of the power spectrum were at integer multiples of the presentation rate.

To measure changes in sound-evoked activity following LDS presentation, peristimulus spike time histograms (PSTHs) of the PRE and POST spike trains were computed (0.5 ms bins), by averaging cycles from each of four 5-s time windows of tone pip presentation (Figure 2A, T1–T4). From these time windows, we quantified the total, spontaneous, and tone-evoked spiking activity. The spontaneous spike rate was estimated from the last 10 ms of the cycle histogram (Figure 2D, red dashed line and red arrow). To estimate the tone-evoked spikes, we first determined the response time window (Figure 2D, blue arrow), based on the time bins that exceeded a criterion threshold of 5% of the peak above the spontaneous rate (threshold rate = 95% * spontaneous rate + 5% * peak rate). To account for noise fluctuations of PSTH bin spike rates, the response time window was extended to the nearest bins before and after the peak that fell below the criterion rate for 2 ms (four consecutive bins). Finally, the evoked spike count was calculated by integrating the PSTH during the response window (Figure 2D, blue shaded area), and subtracting the expected spontaneous spike count (spontaneous count = spontaneous rate x response duration) during this window. For each parameter, we estimated a 95% CI of the baseline from the four 5-s windows PRE-LDS. Changes in response parameters were classified as potentiated or suppressed if the POST measure fell above or below the 95% CI of the same channel, respectively. We quantified these changes by normalizing the difference to the sum of the matching PRE and POST windows [Δ = (POST−PRE)/(POST + PRE)].

To express the relative contribution of evoked spikes to the total spike count, we calculated the evoked fraction as (evoked spikes/total spikes), where a fraction of 1 indicates that the response is completely composed of evoked spikes only, and a fraction of 0 indicates that none of the spikes during the cycle is stimulus evoked. The total spike count is approximately the sum of the evoked and spontaneous spike counts. Thus, the evoked fraction provides a measure of the relative contribution of evoked and spontaneous spikes. Changes of evoked fraction following the LDS are presented as a difference (POST – PRE).

#### Combination of Spontaneous and Tone-Evoked Paradigms

The relationship between spontaneous and tone-evoked changes after LDS was assessed with a linear correlation. Here, we paired spontaneous activity trials to tone-evoked activity trials with matching LDS parameters (sound intensity and center frequency). When multiple trials were repeated with the same parameters, we chose pairs that were the closest in time to each other to reduce the influence of anesthetic state on the results.

### Statistics

All data plots and the corresponding statistical analyses were performed using Origin Pro (OriginLab Corporation, Northampton, MA, United States). For independent samples comparisons, a 1WayANOVA, followed by a Scheffe’s test, was performed. To compare paired samples, we used the Wilcoxon signed rank test. For the correlation analysis a linear correlation was performed, and Pearson’s correlation coefficient was reported. For the comparison of fractions we used a 2-sample proportion comparison with Fisher’s exact test. Statistical significance is stated if the *p*-value is smaller than 0.05. In the figures statistical significance is marked as the following: *p* < 0.05 = *, *p* < 0.01 = **, *p* < 0.001 = ***.

## Results

### Effects of Long-Duration Sound on Spontaneous Activity

We first investigated the effect of an LDS on spontaneous multi-unit activity recorded from the mouse IC. We used 16 or 32-channel probes in 1–3 penetrations per animal to record spontaneous activity beginning 30–60 s before, and until up to 240 s after a 60 s, 1/3 octave, narrow-band, noise LDS that was usually at least 30 dB above the lowest pure tone threshold of that center frequency across all channels (Figure 1). We tested two to three LDS noises at different center frequencies at least 1/2 octave apart in each animal. Among all the recordings in all animals, we observed afterdischarges in 15.5% (346 of 2226) of the channels that were responsive to the LDS.

**FIGURE 3:**
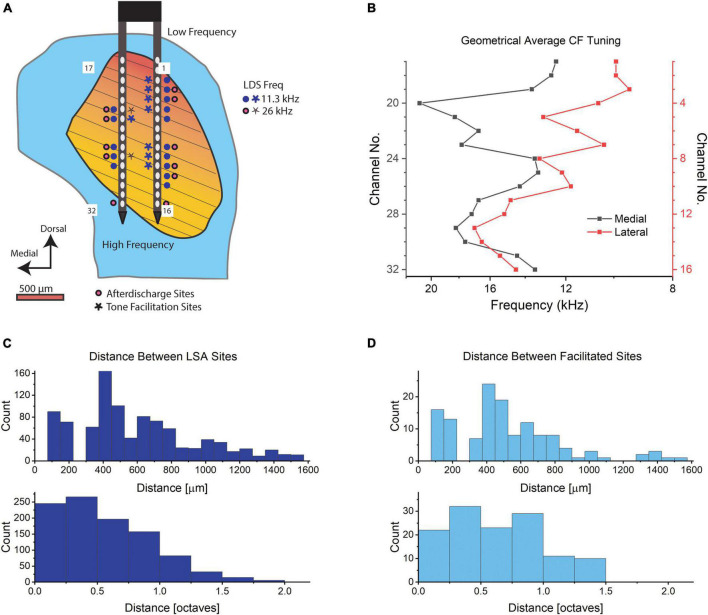
Recording sites in the IC. **(A)** Diagram of an example right IC in a transverse section. The blue indicates the entire IC while the orange to yellow gradient indicates the central nucleus with the orientation of fibrodendritic lamina shown with black lines. A 32-channel electrode is shown with numbered shanks that correspond to the channel number in **(B)**. Potentiated sites from one animal are shown in response to tones at 11.6 kHz LDS (blue) and 26 kHz LDS (red). **(B)** The geometric average CF tuning in kHz for each channel averaged across *n* = 6 mice. Red is the lateral shank (channels no. 1–16) and black is the medial shank (electrodes no. 17–32). **(C)** Histogram of the distance in μm between LSA sites from the same six mice, *n* = 997, upper panel distance in μm, lower panel distance in octaves. **(D)** Histogram of the distance between potentiated sites from six mice, in μm (*n* distances between channels = 131, upper panel) and octaves (*n* distances between channels = 126, lower panel; we were unable to determine the CF of one potentiated channel. Thus, the decreased number of distances in the octave distance calculations compared to the metric distances). Bin size in **(C,D)** upper panel: 75 μm, lower panel: 0.25 octaves.

**FIGURE 4:**
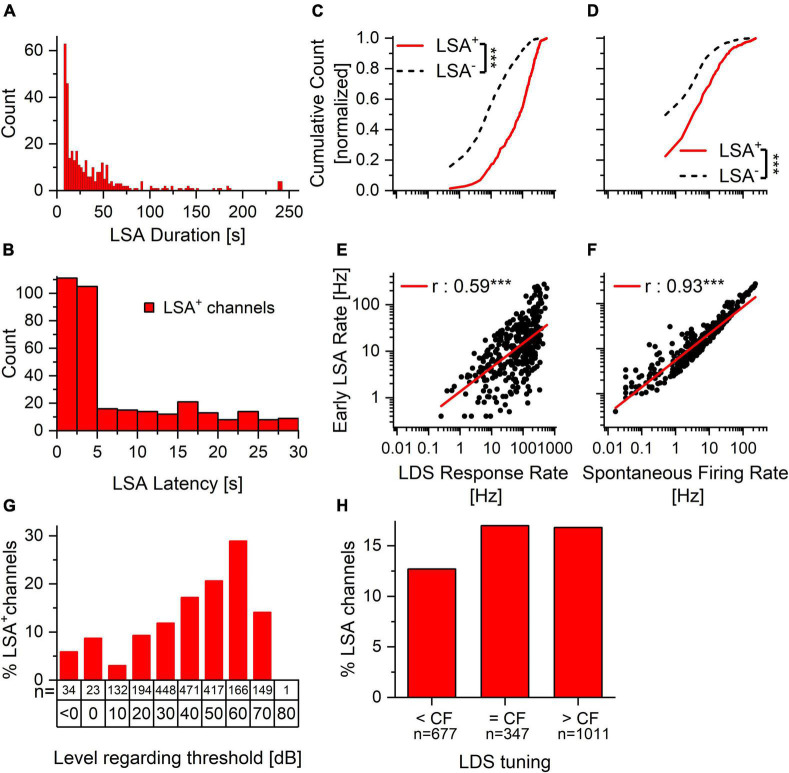
Spontaneous activity and response to the LDS predict the presence and size of afterdischarge activity. The proportion of LSA positive and LSA negative channels is similar in channels tuned to the LDS and tuned above or below. Within the tuned channels the proportion rises with increasing stimulus level. **(A)** LSA duration ranges from seconds to minutes. While the duration of the majority of the LSA is below 1 min, there are several channels with a longer LSA. **(B)** LSA latency is predominately less than 5 s after the offset of the LDS. It can, however, extend to the maximum of our cutoff time (30 s). **(C,D)** Normalized cumulative count of LDS response rate **(C)** and spontaneous firing rate **(D)** of long-duration afterdischarge positive (LSA+, solid red line, *n* = 346) and afterdischarge negative (LSA–, dashed black line, *n* = 1880) channels. **(E,F)** Correlation of the early afterdischarge firing rate to the LDS response rate **(E)** and spontaneous firing rate **(F)** for individual afterdischarge positive (LSA+) channels. ***p < 0.001. **(G)** Percentage of channels with LSA in response to increasing stimulus levels above threshold for channels in which the CF matches the LDS. Only channels responsive to the stimulus and for which the CF could be clearly identified were included in this analysis. **(H)** Percentage of LSA positive channels for experiments with LDS frequency below, at or above the channel’s CF. The relationship between LDS response rate and LDS tuning re CF for both LSA^+^ and LSA^–^ channels can be seen in Supplementary Figure 3A. The relationship between LSA rate and LDS tuning re CF can be seen in Supplementary Figure 3B.

The proportion of channels exhibiting afterdischarges in these experiments is comparable to the proportion of IC neurons with LSA observed in a much smaller sample of single neurons studied with patch pipettes (Ono et al., 2016). The channels where LSA was observed were distributed throughout the IC (Figure 3A and Supplementary Figure 2, filled circles). Since the multi-channel probes span much of the IC, we can see the simultaneous activity throughout a sample of IC neurons with many different characteristic frequencies (CF, Figure 3B). Figure 3C shows the distances between sites with an afterdischarge in response to single stimulus presentations for the recordings shown in Figure 3A and Supplementary Figure 2. Most often there are several channels exhibiting LSA phenomenon in the same penetration that are unlikely to be from a single source that is picked up by adjacent channels. Separations of 400–600 μm were common (Figure 3C, upper panel). The distance between the CFs of channels with LSA is also expressed in octaves (Figure 3C, lower panel). Only 24.5% of channels are tuned within a quarter octave of each other. So, the ∼70 dB SPL LDS can activate LSA in neurons that differ widely in CF. The duration of the recorded afterdischarges ranged between the minimum duration to reach our threshold (7.5 s) to the maximum of our recording length (240 s, 95th percentile: 141.6 s, Figure 4A). The latency of the LSA response was usually 5 s or less but in some cases the LSA latency extended out to our cutoff of 30 s (Figure 4B).

We asked whether the LSA is a stochastic response or is predictable by other factors. As the afterdischarge follows the response to the LDS, it is tempting to propose that the response to the LDS is predictive of the incidence and intensity of the afterdischarge. We presented the LDS at levels approximately 30 dB above lowest observed threshold for the center frequency of the acoustic stimulus being used (30–90 dB SPL). Consequently, the multi-unit response to the LDS varied between channels since the 32 channels were distributed across the IC and responded best to different CFs. Nevertheless, there was a consistent pattern across the samples. Channels with an afterdischarge had higher firing rates in response to the LDS [Figure 4C, one-way ANOVA: *f*(1,2224) = 569.01, *p* < 0.001]. We also found that channels with LSA (LSA^+^) had higher spontaneous firing rates before the LDS presentation (PRE) than channels without LSA [LSA^–^; Figure 4D, solid red vs. dashed black lines, respectively; one-way ANOVA: *f*(1,2224) = 94.58, *p* < 0.001].

When analyzing the relationships between the different LSA parameters (latency, duration and firing rate) via linear correlation we found that while the duration was longer when the latency was shorter (Pearson’s *r* = –0.339, *p* < 0.0001), the latency itself was shorter if the LDS firing rate was higher (*r* = –0.269, *p* < 0.0001). The rate of the LSA was correlated with the duration of the LSA (*r* = 0.1613, *p* = 0.00263). However, it was not correlated with the latency of the LSA onset (*r* = –0.0734, *p* = 0.1732). We then asked whether the strength of the afterdischarge was related to the evoked spike rate during the LDS and/or the spontaneous rate and found that both were significantly correlated with the strength of the afterdischarge (LDS response rate: *r* = 0.57, *p* < 0.001; spontaneous rate: *r* = 0.93, *p* < 0.001). Channels with a larger afterdischarge had both a higher spontaneous rate and responded to the LDS with a higher firing rate (Figures 4E,F). Thus, a higher baseline spiking activity and higher spiking activity evoked by the LDS lead to a higher prevalence of afterdischarge activity, as well as a bigger afterdischarge response, and this suggests a robust phenomenon.

Because sound-driven responses in the IC vary with the spectral composition of the stimulus, we also examined whether the relationship between the center frequency of the LDS and each channel’s characteristic frequency (CF) was predictive of the presence/absence of an LSA. Presence of LSA was not strongly affected by whether the channel’s CF was below, at or above the LDS center frequency (**Figure 4H**). We also asked if the presence of an afterdischarge depended on the level of the LDS above the channel’s threshold. While the LDS was presented usually at a level at least 30 dB above the lowest threshold across all channels, this level could be less than 30 dB above threshold in individual channels with higher thresholds. We found that the LSA was more prevalent with increasing stimulus intensity above threshold at the CF (Figure 4G).

### Tone Evoked Activity

To observe changes in tone pip driven activity after an LDS presentation, we recorded responses to 3 ms pure tone pips as depicted in Figure 2. We made recordings from 32-channel probes in each animal in which tone pip trials were repeated using different center frequencies about one octave apart. Tone pips were presented at a presentation rate of 21 Hz, and at a level 20–30 dB above the lowest threshold observed in any channel responding to the frequency of the tone being presented. Similar to the presentation of tones used to evoke auditory brainstem responses, the 3 ms tone pips at a high presentation rate (21 Hz) allowed us to collect many tone-evoked responses in a short length of time. The tone pips were broken into four trains of 5 s duration separated by 5 s silent gaps (Figure 2, *n* = 6 animals). For one of the animals, six tone pip trains were presented. The silent gaps were necessary to establish the baseline response of the neurons prior to the LDS since continuous long-durations trains of noise bursts have been shown to elicit an afterdischarge that continues after the offset of the train. The 5-s-on/5-s off tone pip trains were repeated after a 60 s LDS (narrow band noise, 0.33 octave bandwidth), whose center frequency matched that of the pure tone pips, and whose sound level was 30 dB above the lowest pure tone threshold of that center frequency. There was a silent gap (1–2 s) between LDS and POST-LDS tone pips. An example of suppressed responses after the LDS is shown in Figure 2B (downward pink arrow indicates a shift in the tone evoked activity), while Figure 2C illustrates a channel with potentiated responses to tones. In this channel, it is clear that spike rates during silent (green dashed line) as well as stimulus presentation periods (magenta dotted line) are increased following the LDS presentation compared to baseline (see upward green and pink arrows indicating the shift in spontaneous and tone evoked activity, respectively). To better examine these changes, we computed cycle-averaged peristimulus time histograms (PSTHs), and quantified different aspects of the tone pip responses: the total spikes, the evoked spikes, the spontaneous spikes and the relative contribution of the evoked spikes to the overall spikes (Figure 2D, also see methods).

**FIGURE 5:**
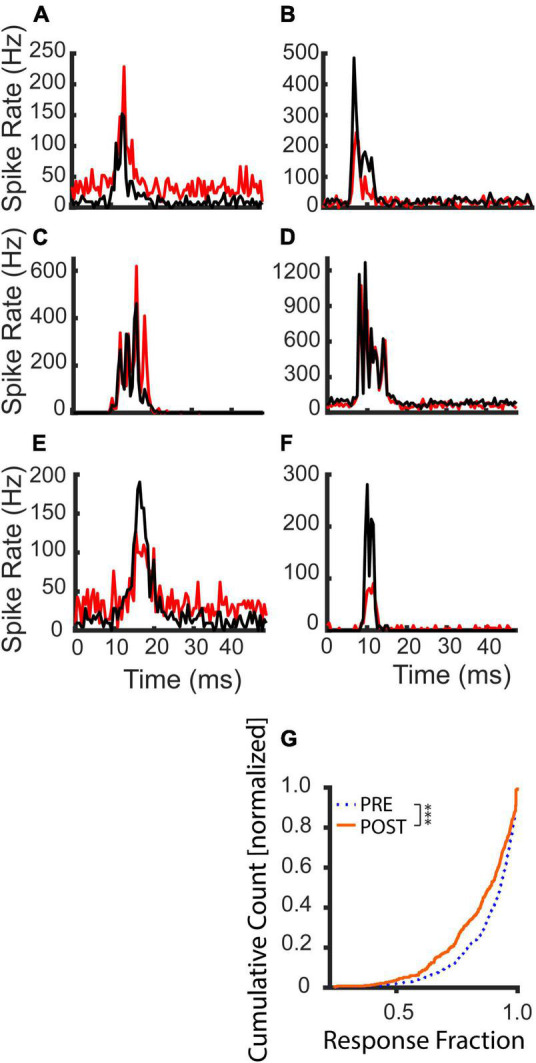
Examples of tone evoked response changes following the LDS and cumulative distribution of the evoked fraction before and after an LDS presentation. Black lines indicate PRE-LDS cycle-averaged peristimulus spike time histograms (PSTHs), red lines POST-LDS PSTHs (0.5 ms bins). **(A,C,E)** Responses with potentiated average spike rates. **(B,D,F)** Responses with suppressed average spike rates. **(G)** Comparison of the evoked fraction (contribution of the tone evoked response to the overall activity in the cycle) between PRE and POST T1 analysis windows (PRE – dotted blue line; POST – solid orange line, *n* = 367). The statistical comparison was done with a paired-sample Wilcoxon signed rank test. ***p < 0.001.

Figure 5 illustrates example PSTHs of tone pip responses before (black) and after (red) the LDS. Following the LDS, average spike rates could be increased (Figures 5A,C,E) or decreased (Figures 5B,D,F). In some channels, these changes could reflect concurrent increases or decreases of the evoked and spontaneous activity (Figures 5A,B). However, overall spike rate changes did not always fully capture underlying changes in evoked and spontaneous activity. Figure 5C shows an example where the spike rate increase is mediated solely by increased evoked spiking, and Figure 5D illustrates spike rate depression mediated solely by decreased spontaneous spiking. Responses could entail decreased evoked spiking despite increased spontaneous spiking (Figure 5E), or the evoked response could decrease without a change in spontaneous activity (Figure 5F). These examples demonstrate that tone pip responses can undergo robust changes of evoked and/or spontaneous activity following LDS presentation.

Response onsets latencies typically varied between 6 and 8 ms, since each channel was at a different depth in the IC and had a different multi-unit CF (Figures 3A,B). Interestingly, the response durations were an average of 11.7 ms (±0.45 SEM). These were considerably longer than the duration of the 3 ms tone pip stimulus.

We examined multi-unit activity at 375 stimulus responsive recording sites and found a subpopulation of those channels (15.3%) that had a significant increase in total spike rate immediately after the LDS in contrast to the more expected reduced spike rates (48% were significantly reduced). Across all channels a slight, but significant decrease in total spiking was observed [paired-sample *t*-test; PRE: 44.7 Hz ± 41.1, T1 POST: 42.7 Hz ± 43.8, *t*(374):3.1, *p* = 0.002]. Channels with facilitation (Figure 3A and Supplementary Figure 2, stars) were distributed across the penetration site and were often at the same locations as for the afterdischarges (Figure 3A and Supplementary Figure 2, filled circles). The distribution of distances between recording sites with facilitation also indicates that it was not a localized phenomenon (Figure 3D). This shows a very similar distribution pattern for the distance in μm to the LSA^+^ sites. Their tuning distances is narrower (up to 2 octaves for LSA^+^ vs. up to 1.5 octaves for facilitated sites). However, only 17.3% of facilitated responses are tuned within a quarter octave of the LDS frequency, in comparison to 24.5% of LSA^+^ channels tuned within a quarter octave of the LDS frequency (Figures 3C,D, respectively).

Both changes of spontaneous and evoked activity could contribute to the observed changes in spike rate. Evoked activity composed the majority of the spikes in most channels, but this fraction generally decreased after the LDS (Figure 5G). While total spike rates increased in 15.3% of channels, significant increases of evoked fraction were only seen in 7.1% of channels. This can be explained by the finding that spontaneous activity was frequently facilitated [significant facilitation in 41.4% of channels; PRE: 10.6 ± 26.5 Hz, POST T1: 14.7 ± 30.1 Hz paired-sample *t*-test: *t*(374): –5.2, *p* < 0.001], while evoked activity was generally suppressed [significant facilitation in 9.4% of channels; PRE: 198.1 ± 166.5 Hz, POST T1: 178.7 ± 168.4 Hz paired-sample *t*-test: *t*(365): 6.39, *p* < 0.001], following the LDS.

**FIGURE 6:**
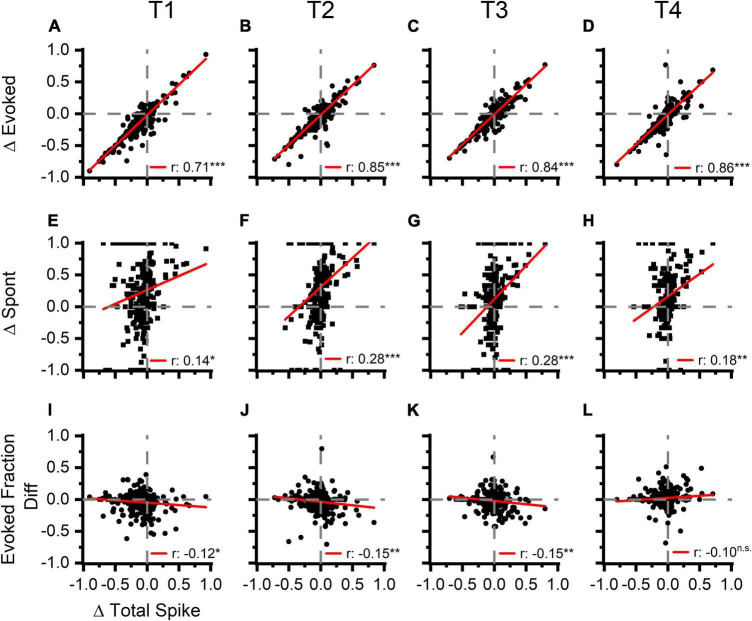
Increases in tone evoked activity is strongly correlated with an increase total spike rate following LDS presentation. Spontaneous activity and evoked fraction are also correlated to the overall total spike rate but to a lesser extent. First Row **(A–D)**: the correlation of normalized PRE-POST differences of tone evoked activity (Δ Evoked) and overall firing rate (Δ Total Spike). The four columns represent the four consecutive time blocks (5 s each as in marked in Figure 2). Second Row **(E–H)**: the correlation of normalized PRE-POST difference of the spontaneous activity (Δ Spont) and Δ Total Spike. Third Row **(I–L)**: the correlation of PRE-POST differences of the Evoked Fraction (Evoked Fraction Diff) and Δ Total Spike. Normalized difference was calculated thus: (POST-PRE)/(POST + PRE), *n* = 6 (animals). Red lines represent linear correlation.

To track these changes over time, we measured total, evoked, and spontaneous spikes in four 5 s analysis windows (Figure 2A, T1–T4, up to 35 s post LDS offset). Across all four windows, the PRE/POST differences in evoked spiking (△ Evoked, Figures 6A–D) were strongly correlated with differences in total spike rate (△ Total Spike, Figures 6A–D and Table 1). We also examined whether the directions of change in overall, evoked, and spontaneous spiking persist across the successive time windows. Table 2 shows that the distribution of channels across the quadrant’s changes over time with the largest change being observed between T1 and T2. To describe whether individual channels have persistent changes of total and evoked spiking, we compared which quadrant of the Δ evoked vs. Δ total spikes function (Figures 6A–D) the channel’s response appeared in, across the four time windows. Seventy percent of the channels remained in the same quadrant in greater than 50% of the time windows. Most of these channels (73%) showed decreased evoked and total spikes, while 26% of these channels showed increased total spikes (21.7% with an increase in evoked spikes and 3.8% with a decrease in evoked spikes).

**TABLE 1:** Linear correlation of different stimulus evoked response parameters.

	Δ Evoked Spikes vs. Δ Total Spikes	Δ Spontaneous Spikes vs. Δ Total Spikes	Evoked Fraction Difference vs. Δ Total Spikes
T1	*R* = 0.71, *p* < 0.001	*R* = 0.14, *p* < 0.013	*R* = –0.12, *p* < 0.026
T2	*R* = 0.85, *p* < 0.001	*R* = 0.28, *p* < 0.001	*R* = –0.15, *p* < 0.003
T3	*R* = 0.84, *p* < 0.001	*R* = 0.28, *p* < 0.001	*R* = –0.15, *p* < 0.004
T4	*R* = 0.86, *p* < 0.001	*R* = 0.18, *p* < 0.003	*R* = –0.10, *p* < 0.060

	**Δ Total Spikes** **vs.** **LDS Response Rate**	**Δ Evoked Spikes** **vs.** **LDS Response Rate**	**Δ Spontaneous Spikes** **vs.** **LDS Response Rate**

T1	*R* = 0.38, *p* < 0.001	*R* = 0.24, *p* < 0.001	*R* = 0.36, *p* < 0.001
T2	*R* = 0.24, *p* < 0.001	*R* = 0.20, *p* < 0.001	*R* = 0.13, *p* = 0.028
T3	*R* = 0.33, *p* < 0.001	*R* = 0.26, *p* < 0.001	*R* = 0.09, *p* = 0.135
T4	*R* = 0.17, *p* < 0.001	*R* = 0.20, *p* < 0.001	*R* = 0.17, *p* = 0.006

**FIGURE 7:**
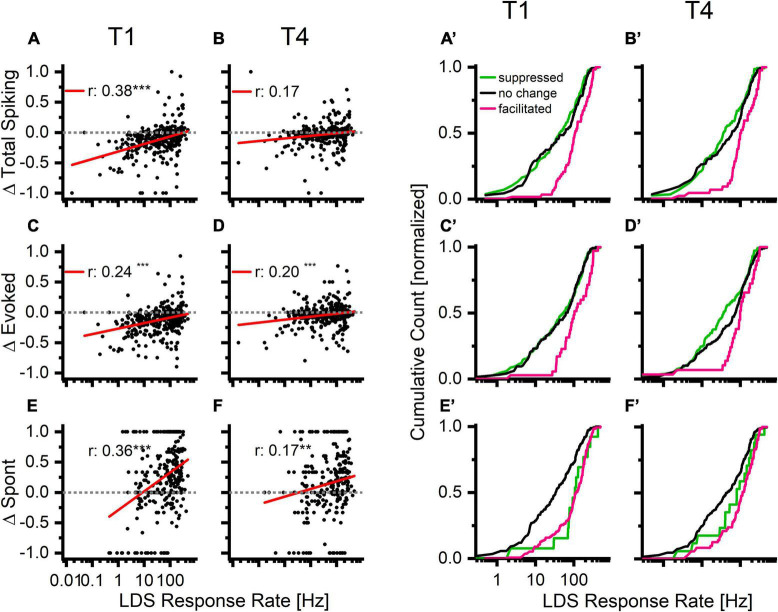
Increases in overall firing, tone driven, and spontaneous activity occur more often in channels with higher firing rates in response to the long duration sound. Top row: normalized PRE-LDS vs. POST-LDS differences in the overall firing rate (Δ Total Spiking) compared to response to the LDS response rate. Middle row: normalized PRE-LDS vs. POST-LDS differences in the tone evoked response (Δ Evoked) compared to response to LDS. Bottom row: normalized PRE-LDS vs. POST-LDS differences in the spontaneous activity (Δ Spont) compared to response to LDS. Two left columns: The individual data points in the first and last analysis window (**A,C,E**: T1; **B,D,F**: T4). Two right columns: The same data as a cumulative count that is organized by statistical facilitation/decrease/no change to baseline, defined as either exceeding the 95% confidence interval (CL) of the baseline or being below the 5% CL of the baseline (**A’,C’,E’**: T1; **B’,D’,F’**:T4). Color coding is based on statistical analysis of the corresponding parameter (pink-facilitation > 95% CL, black-unchanged, green-suppressed < 5% CL).

***Upper half**: correlation coefficients between the difference in total spiking changes and the difference of evoked spikes, spontaneous spikes, evoked fraction across the four analysis windows (T1–T4). **Lower half**: Correlation coefficients between the difference in LDS rate and difference in total, evoked, and spontaneous spiking changes. T1–T4 indicate the analysis window. R values represent Pearson’s R. Linear correlation of Δ Evoked, Δ Spontaneous and Δ Evoked Fraction to Δ Total Spikes represent the data in Figure 6. The correlation analysis of all stimulus driven response parameters to LDS response rate represent Figures 7A–F and additionally from analysis windows T2 and T3.*

**TABLE 2:** Quadrant analysis.

	Δ Evoked Spikes vs. Δ Total Spikes	Δ Spontaneous Spikes vs. Δ Total Spikes	Evoked Fraction Difference vs. Δ Total Spikes
T1	UL: 5.87% UR: 13.33% LL: 70.93% LR: 9.87%	UL: 58.13% UR: 21.07% LL: 18.67% LR: 2.13%	UL: 30.93% UR: 6.67% LL: 45.87% LR: 16.53%
T2	UL: 3.20% UR: 30.13% LL: 56.27% LR: 10.40%	UL: 45.07% UR: 35.73% LL: 14.40% LR: 4.80%	UL: 25.07% UR: 16.00% LL: 34.40% LR: 24.53%
T3	UL: 6.93% UR: 31.47% LL: 52.00% LR: 9.60%	UL: 43.73% UR: 32.53% LL: 15.20% LR: 8.53%	UL: 28.53% UR: 14.40% LL: 30.40% LR: 26.67%
T4	UL: 6.93% UR: 31.47% LL: 52.27% LR: 9.33%	UL: 42.67% UR: 35.20% LL: 16.53% LR: 5.60%	UL: 27.20% UR: 15.73% LL: 32.00% LR: 25.07%

*Analysis of distribution of channels in the four quadrants of Figure 6.*

*UR: Increase in both parameters (total spikes and evoked spikes/spontaneous spikes/evoked fraction), LL: decrease in both parameters, UL: decrease in total spikes and increase in the other parameter, LR: Increase in total spikes and decrease in the second parameter.*

*T1–T4 indicate the analysis window. n = 357 channels.*

Spontaneous activity PRE/POST differences (Figures 6E–H, △ Spont) were also correlated with the differences in total spiking activity; however, the linear relationship was much weaker than observed for the evoked activity changes (Table 1). This is in part explained by the finding that evoked spikes compose the majority of the spikes in most channels (Figure 5G), resulting in a high covariance between evoked and total spike changes. Additionally, the relationship between spontaneous spiking changes with total spiking changes may be inadequately described with a linear model, as this relationship is different between channels with and without LSA (Supplementary Figure 4). Changes were also less persistent than changes of evoked and total spikes, as only 50% of channels had changes that persisted for the majority of the time windows, with the majority (57%) of these channels showing an increase in spontaneous activity without increase of total spiking (upper left quadrant, Figures 6E–H).

Changes in the fraction of the response due to the evoked spikes (evoked fraction) were weakly and negatively correlated with the total spiking changes (Figures 6I–L and Table 1). While increases of total spiking were typically associated with increases of the evoked spikes (Figures 6A–D), they were also associated with more robust increases of spontaneous spikes (Figures 6E–H), resulting in a slight reduction of evoked fraction. Thus, regardless of whether the overall spike rate increased or decreased, the relative contribution of evoked and spontaneous spikes was consistent. This supports the hypothesis that increased spontaneous/afterdischarge activity contributes to changes of tone-pip evoked responses in channels with increased spiking activity after the LDS.

About half (49%) of the channels showed changes of evoked fraction and total spiking that persisted in the same quadrant more than 50% of analysis windows. As with the assessment of evoked and total spiking changes, the most channels (51%) fell in the lower left quadrant.

Because the afterdischarge strength was positively correlated with the strength of the response to the LDS (Figure 4E), we hypothesized that the response to the LDS might also predict differences in tone-evoked responses. Indeed, channels with stronger LDS responses were somewhat more likely to increase overall spike rate after LDS (Figure 7A). This relationship was strongest in the first 5 s analysis window (T1) and weakened in successive windows (Figures 7A,B and Table 1, bottom half).

As further evidence of the relationship between LDS response strength and tone pip response rate changes, the LDS response rate was significantly different between channels with significant increases (facilitation, magenta), decreases (suppression, green), or no change (black) from baseline [Figures 7A’,B’, one-Way ANOVA, T1: *f*(2,372) = 15.9, *p* < 0.001; T4: *f*(2,372) = 10.5, *p* < 0.001]. The facilitated channels had a significantly higher LDS response rate than the other channels across all analyzed time windows T1–T4 [mean LDS rates in Hz: T1 – facilitated (Fa):146.9, no change (NC): 84.8, suppressed (S):73.98; T2 – Fa: 140.4, NC: 76.1, S: 81.6; T3 – Fa: 135.5, NC: 81.8, S: 78.7; T4 – Fa: 144.2, NC: 87.9, S: 73.96; for statistical results see Table 3].

**TABLE 3:** Scheffe test results for direction of change.

		*Total Spikes*	*Evoked Spikes*	*Spontaneous Spikes*
T1	Suppressed vs. No Change:	*p* = 0.535	*p* = 0.998	*p* = 0.013
	Suppressed vs. Facilitated:	*p* < 0.001	*p* < 0.001	*p* = 0.962
	No Change vs. Facilitated:	*p* < 0.001	*p* < 0.001	*p* < 0.001
T2	Suppressed vs. No Change:	*p* = 0.858	*p* = 0.743	*p* = 0.336
	Suppressed vs. Facilitated:	*p* < 0.001	*p* = 0.003	*p* = 0.812
	No Change vs. Facilitated:	*p* < 0.001	*p* < 0.001	*p* < 0.001
T3	Suppressed vs. No Change:	*p* = 0.952	*p* = 0.923	*p* = 0.349
	Suppressed vs. Facilitated:	*p* < 0.001	*p* = 0.013	*p* = 0.854
	No Change vs. Facilitated:	*p* < 0.001	*p* = 0.004	*p* = 0.007
T4	Suppressed vs. No Change:	*p* = 0.348	*p* = 0.215	*p* = 0.245
	Suppressed vs. Facilitated:	*p* < 0.001	*p* = 0.010	*p* = 0.723
	No Change vs. Facilitated:	*p* < 0.001	*p* = 0.102	*p* < 0.001

*Scheffe post hoc test of LDS response rate with direction of change as the main effect. The test was only performed if the one-Way ANOVA was significant (factor: direction of change). Directions of change are facilitation, no change and suppression (magenta, black, and green in Figure 7 right columns, respectively). n = 375 channels.*

*T1–T4 indicate the analysis window.*

A positive correlation was also observed between changes of evoked activity and the LDS response rate (Figures 7C,D). Again significant differences in LDS response rate were found between channels with facilitation, suppression, or no change in response strength (Figures 7C’,D’, T1 – Fa: 152.2, NC: 83.3, S: 83.9; T2 - P: 138.6, NC: 79.6, S: 87.3; T3 – Fa: 131.5, NC: 82.2, S: 86.1; T4 – Fa: 130.5, NC: 93.2, S: 76.3; for statistical results see Table 3).

Changes in spontaneous activity were positively correlated with changes of LDS response rate (Figures 7E,F). Significant relationships were observed in all analysis windows, though the strongest correlation was observed in the first analysis window (see Table 1, lower half). Interestingly channels that showed no significant change in either direction had significant lower response rates to the LDS presentation than either suppressed or facilitated channels (Figures 7E’,F’, T1 – Fa: 130.9, NC: 65.4, S: 137.8; T2 – Fa: 101.7, NC: 75.4, S: 106.0; T3 – Fa: 114.0, NC: 78.5, S: 103.7; T4 – Fa: 134.9, NC: 78.3, S: 115.7, for statistical results see Table 3). This indicates that a stronger response to the LDS leads to a stronger change in spontaneous activity in either direction.

We find similar results when analyzing the same three parameters (Total spike, evoked spikes and spontaneous spikes) as a function of the relationship between the response to the LDS and the overall PRE activity (Supplementary Figures 3F–H). While the facilitated channels have a higher response rate to the LDS than the no change or suppressed group, the overall PRE firing rate does not show such a clear difference between the groups.

All together, these data indicate that changes of tone pip responses following the LDS depend on the amount of activity driven by the LDS. As the LDS drives higher response rates, the channel is more likely to show facilitation of evoked and total spiking in the subsequent tone pip responses. In contrast, the response to an LDS presentation only predicts a change but not necessarily the direction of it for spontaneous activity.

**FIGURE 8:**
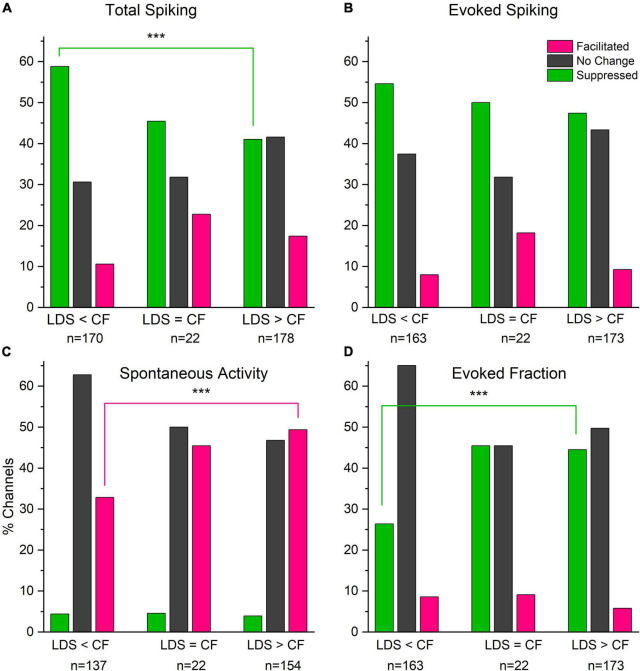
Facilitation of total spike rate and tone evoked response (fraction) is more likely in channels tuned to frequencies that match the LDS frequency than tuned above or below. Spontaneous activity shows very little suppression irrespectively of the relationship of the channel’s CF and the LDS frequency. **(A)** Changes in total spiking activity (Total Spiking). **(B)** Changes in Tone evoked activity (Evoked Spiking). **(C)** Changes in spontaneous activity (Spontaneous Activity). **(D)** Changes evoked fraction (Evoked Fraction). Colors indicate direction of changes (pink – facilitated, dark gray – unchanged, green – suppressed). ***p < 0.001.

We also investigated how the spectral tuning affected the changes of sound-evoked responses, e.g., were the CFs below, at and above the LDS frequency. In channels tuned to the LDS frequency, the total and evoked spiking were more frequently facilitated (Figure 8). Interestingly, spectral tuning has a greater effect on the proportion of facilitated channels, than the proportion of LSA channels (Figure 4H). Potentiation of spontaneous activity was significantly more likely in channels tuned below the LDS frequency than channels tuned above the LDS frequency (Figure 8C, magenta bars, *p* = 0.0037). Suppression of total spiking was most likely in channels tuned above the LDS frequency, where suppression of evoked fraction was least likely [green bars; total spiking (8A) *p* = 0.0007; evoked fraction (8D) *p* = 0.0004]. However, the fraction of channels with total or evoked spiking facilitation was not significantly different across the three different spectral tuning categories. Doing a linear correlation between the normalized difference of total spiking, evoked spiking and spontaneous spiking (Supplementary Figures 3C–E) we found no significant correlation between the size of the change and the tuning for the facilitated channels. For channels with no change, we found a significant correlation only for the total spiking (*r*: 0.227, *p* < 0.01). The suppressed channels, however, did show a significant linear correlation between all three parameters and the tuning of the channels (total spiking *r*: 0.31, *p* < 0.001; evoked spiking *r*: 0.24, *p* < 0.001; spontaneous spiking *r*: 0.18, *p* < 0.001). These data suggest that changes of tone-evoked responses following LDS depend to some extend on the LDS frequency relative to the channel’s spectral tuning.

### Relationship Between Long-Duration Sound Evoked Afterdischarge and Changes in Stimulus Driven Activity

We asked whether the LSA was related to changes in the tone pip evoked responses. To address this question, we matched LSA and tone pip responses from separate recordings in the same channel from the same electrode penetration site, using identical LDS stimuli. We examined the relationship between the amount of change in the afterdischarge and tone pip responses within the same channel. Recordings of LSA were matched to recordings of tone-evoked responses that were closest in time, if multiple trials were recorded. We analyzed 181 channels from paired recordings of 13 LDS stimuli in six animals. Channels with a facilitation of total spiking during tone pip presentation often also had LSA (Figure 3A and Supplementary Figure 2). Of the 26 channels with facilitation in total spikes in the T1 analysis window 73% also were positive for LSA. Including channels not significantly facilitated in total spikes during the tone presentation, 55 channels were LSA^+^ channels (11 LDS stimuli, 6 animals). All data presented were measured from the first analysis time window (T1). We expressed changes of tone pip total, evoked, and spontaneous spikes as a raw spike count difference (POST – PRE), to keep the measurement unit equivalent between the axes.

**FIGURE 9:**
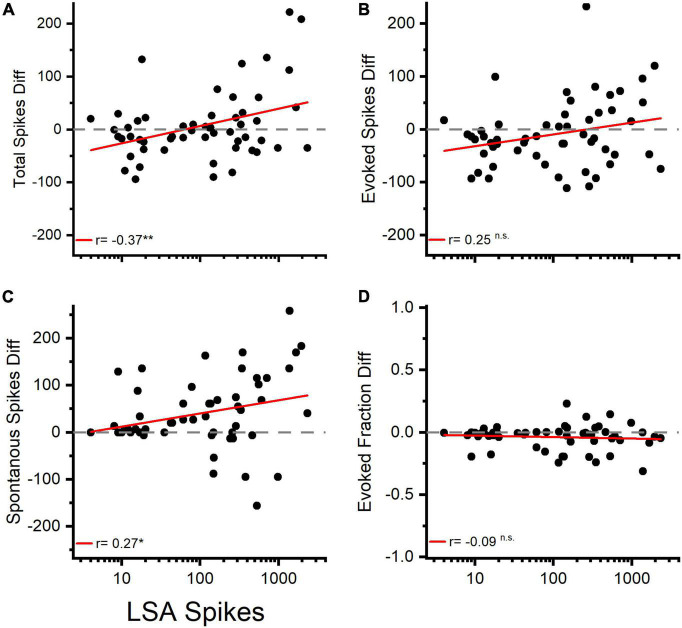
Changes in total spiking and spontaneous activity are correlated with the amount of LSA activity following a matching LDS. **(A)** Changes in Total Spiking activity (Total Spiking Diff) in response to the LSA spike count measured in matching spontaneous activity experiments. **(B)** Changes in tone evoked response in correlation to the previously observed LSA activity. **(C)** Changes in spontaneous activity in correlation of the previously recorded LSA activity. **(D)** Changes in the evoked fraction (EvokedF) vs. the amount of previously recorded LSA activity. *n* = 55 channels. Red lines represent linear regression analysis.

**FIGURE 10:**
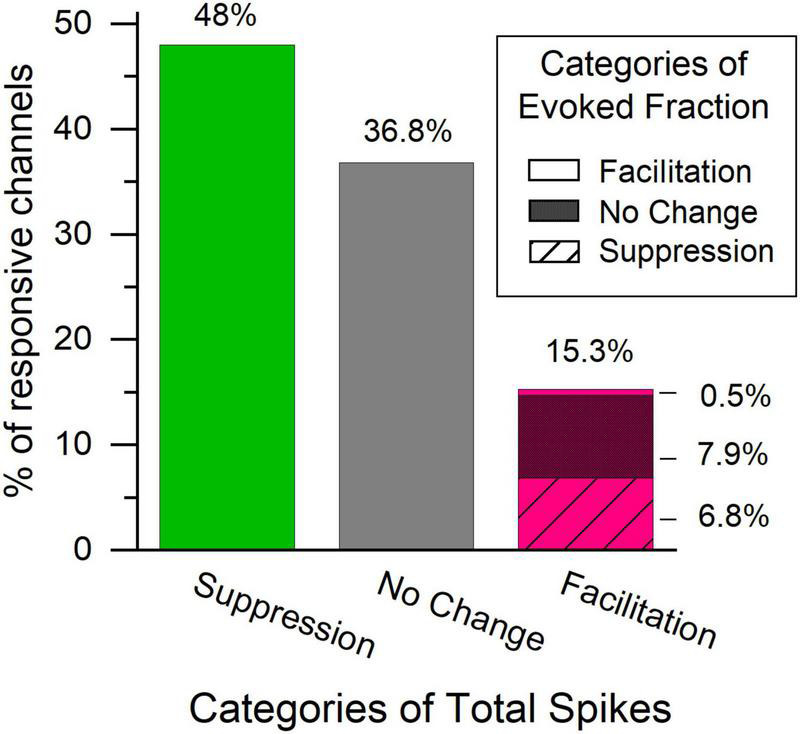
The majority of channels with facilitated total spike rate during the POST tone pip presentation have an unchanged or suppressed evoked fraction in the first analysis window. Bars represent the distribution of direction of change in the overall spiking activity in the first analysis window following a LDS presentation. Green – suppressed total spiking activity, gray – no change in total spiking activity, Magenta – facilitation. Pattern in the magenta bar represent changes in the evoked fraction within the channels with facilitated total spiking activity. No pattern – facilitated evoked fraction, dense crosshatch – no change evoked fraction, striped – Suppressed evoked fraction, *n* = 367.

The total spike count of the afterdischarge showed a positive correlation with the difference in total spikes during the tone pip presentation (Figure 9A), while it showed a less pronounced correlation with the change in spontaneous activity (Figure 9C), and no significant correlation with differences in evoked spikes or evoked fraction (Figures 9B,D and Table 4). This supports the idea that afterdischarges contribute to the increased overall firing rates during sound-evoked responses. As illustrated in Figure 2C, the response to tones after an LDS may have a higher spike rate because it is riding on increased background spiking activity that is not driven by the acoustic stimulus. Although about 15% of channels show a facilitation in spike rate immediately following an LDS, most of those channels have an unchanged or even suppressed evoked fraction (Figure 10). This further supports the notion that the potentiation of tone pip spike responses is predominantly due to an increase in background spiking activity, with little contribution of increased evoked spiking.

**TABLE 4:** Linear correlation of stimulus driven vs. spontaneous activity.

	Total Spikes vs. LSA Count	Evoked Spikes vs. LSA Count	Spontaneous Spikes vs. LSA Count	Evoked Fraction vs. LSA Count
T1	*R* = 0.37, *p* = 0.005	*R* = 0.25, *p* = 0.060	*R* = 0.27, *p* = 0.046	*R* = –0.09, *p* = 0.526
T2	*R* = 0.48, *p* < 0.001	*R* = 0.17, *p* = 0.211	*R* = 0.49, *p* < 0.001	*R* = –0.38, *p* = 0.004
T3	*R* = 0.47, *p* < 0.001	*R* = 0.40, *p* = 0.002	*R* = 0.40, *p* = 0.002	*R* = –0.23, *p* = 0.092
T4	*R* = 0.33, *p* = 0.013	*R* = –0.07, *p* = 0.616	*R* = 0.42, *p* = 0.001	*R* = –0.26, *p* = 0.056

*Correlation coefficients between LSA spike count and changes in tone evoked response parameters (difference (POST-PRE) in total spikes, evoked spikes, spontaneous spikes, and evoked fraction) from matched channels and the same LDS parameters. Data from T1 is illustrated in Figure 9. n = 55 channels (6 animals, 11 different matched experiments).*

## Discussion

Our findings describe a subset of multi-unit recordings in the central nucleus of the IC that show a LDS-evoked facilitation, a significant increase in firing rate following the offset of an LDS both in silence and in response to tone pips. This LDS-evoked facilitation was found at ∼16% of responsive channels in the IC. Both afterdischarges and facilitated firing rates during tone pip responses were associated with stronger responses to the LDS. Facilitation during tone pip responses was rarely accompanied by increased evoked fraction, rather it was more typically accompanied by no change or suppression of evoked fraction (Figure 10, magenta bar). Thus, the facilitation of the sound-evoked response after an LDS is most often due to an increase in spontaneous activity upon which the stimulus-locked response is riding, or a general amplification of both evoked and spontaneous spiking.

We assume that these recordings usually reflect the activity of multiple neurons at each channel. Multi-unit recording is unavoidable with the use of methods that allow large samples of neurons to be recorded simultaneously. Furthermore, the 3 ms tone pips studied here result in many action potentials overlapped in time, making accurate computational spike sorting impossible. However, the interpretation of the present results is aided and clarified by our previous study using patch pipettes to isolate single neurons in the IC and characterize afterdischarges following a LDS (Ono et al., 2016). That study identified single neurons with little or no spontaneous activity that continued to fire after the offset of a long duration sound. These were “sustained regular” neurons (Sivaramakrishnan and Oliver, 2001) whose afterdischarge was positively correlated with the duration and intensity of the LDS. In the present recordings the response at any one channel may represent multiple neurons that respond differently to a LDS. Overall, 16% of channels had a significant increase in spontaneous firing or facilitation of sound-evoked responses. On one hand this facilitated activity at one channel might be the result of a facilitated response of the majority of the neurons recorded at this channel. On the other hand, it is possible that the presence of a few, but high-rate potentiated neurons shift the whole multi-unit activity above our criteria of significant increased activity. Certainly, there may be instances of a facilitated channel including a neuron that was silent before a LDS but produced an afterdischarge, that is spontaneous activity, after the offset of the LDS. Additionally, adapting neurons may be next to neurons with increased responses resulting in an average tone pip response in a channel that is below our threshold for significant facilitation. Since the multiunit response combines these units, it may lead to an underestimation of the number of neurons whose response is enhanced by a LDS. The finding of channels with afterdischarges or facilitated responses to tone pips is thus despite factors that would seem to be unfavorable.

Spike rates during tone pip presentation usually were suppressed following the LDS (nearly half of the responsive channels) or unchanged. Some spike rate suppression is to be expected after the LDS, due to multiple adaptation mechanisms in the auditory pathway (Westerman and Smith, 1984; Ingham and McAlpine, 2004; Malmierca et al., 2009). The vigorous firing during an LDS is likely to cause depletion of synaptic vesicle pools in the cochlea and elsewhere in the auditory pathway leading to the IC. If inputs to the IC are reduced after the LDS, it could result in a momentary reduction in input driven activity. Since our LDS stimuli are considerably less intense and shorter in duration than sounds usually used to produce a temporary threshold shift (TTS) (Lonsbury-Martin and Martin, 1981; Amanipour et al., 2018; Fernandez et al., 2020), we consider a TTS due to transient cochlear damage to be unlikely. Another argument against peripheral damage is that the afterdischarges or facilitation usually subside in a matter of minutes, unlike the typical TTS. However, sensory adaptation mechanisms in the central auditory system may be a factor in situations that produce sound-induced facilitation, and hearing could be briefly altered. This is discussed further below.

Another factor in this study is that all experiments were conducted on anesthetized animals. Isoflurane is known to reduce the amplitude of auditory brainstem responses (Stronks et al., 2010) or reduce the spiking activity at the level of the auditory cortex (Noda and Takahashi, 2015). LSA and the observed facilitation are examples of increased responses, the opposite of the expected effect of anesthesia. In the study by Ono et al. (2016) LSA phenomenon was recorded not only in the IC of mice anesthetized with isoflurane, but also in the IC of urethane anesthetized mice. Additionally, M. Ono was able to record LSA in single units also in awake preparations (February 20–24, Ono et al., 2016). While anesthesia still might play a role in overall neuronal activity the anesthetic state, our PRE recording phase and the POST recording phase should be relatively similar due to their close relation in time. It is therefore unlikely that the here reported phenomenon of LSA and facilitation following an LDS presentation is due to isoflurane anesthesia.

### Timing of Facilitation by Long Duration Sound

Synaptic plasticity is often described in terms of time frame, e.g., short-term or long-term. Both can manifest as either a facilitation or depression of activity. While LTP or long-term depression (LTD) has been implicated in memory formation and learning and usually lasts hours to days (if not longer), short term plasticity is thought to be a reversible mechanism with a time frame in the ms to min range (Friauf et al., 2015). Short term plasticity can be seen in the ms range, but it can also be seen in the seconds to minutes range as either a post-tetanic potentiation (PTP), facilitation, or an equally short depression (Regehr, 2012). The presence of PTP in the auditory system was described several decades ago (Hughes, 1958; Friauf et al., 2015). More recently, Wu et al. (2002) showed that some IC neurons had PTP as well as LTP and LTD. Of course, the LTP *in vitro* experiments in slices were performed with blockers for inhibitory neurotransmitter, so the time course of the events there may be longer than what is seen *in vivo*.

Our *in vivo* multi-unit recordings showed facilitation lasting from several seconds to minutes similar to the LDS-evoked afterdischarges described by Ono et al. (2016) in single neurons. The time course is, therefore, most comparable to the time course of PTP. Also comparable is the use of a LDS as a source of stimulation, much like the tetanizing train of shocks used to induce PTP and LTP or depression *in vitro*.

### Possible Sources of Long-Duration Sound-Induced Facilitation

The mechanisms that produce LDS-induced facilitation are not known. The IC is a major hub in auditory processing and is innervated both from ascending projections from the brainstem as well as from descending projections from the auditory cortex as well as inputs from the contralateral IC (Malmierca, 2004). Although there are numerous inputs to the IC, there is a segregation of their termination sites in the IC. In general, the lemniscal inputs primarily terminate in the central nucleus, while the neocortical inputs primarily terminate in the dorsal and lateral cortex of IC (Chen et al., 2018). Even within the central nucleus, different regions are targeted by different brainstem inputs, some with little overlap (Loftus et al., 2010; Grana et al., 2017). Some inputs are limited by the range of frequencies they represent, while others synapse on only part of a fibro-dendritic lamina leaving other parts of that lamina to other inputs. Our data showed that channels with LDS-induced facilitation were often surrounded by channels without LDS-induced facilitation, but facilitated activity in adjacent channels was occasionally observed (Figure 3). We cannot exclude the possibility that in some cases we were recording activity from the same facilitated neuron(s) with neighboring channels. Although it is possible that facilitation may be driven by a specific input to the IC, it seems unlikely to be restricted to a single area due to the widespread distribution of LSA and tone-evoked facilitation in ICC. Moreover, if a brainstem input is responsible for LSA, it would necessarily have to be driven well by moderate to loud sounds and use glutamate as its neurotransmitter. Thus, inputs to ICC from the contralateral cochlear nucleus and contralateral lateral superior olive seem the most likely candidates.

Long-duration sound-induced facilitation may be generated by mechanisms intrinsic to IC neurons. Neurons in the IC can be divided by morphology (Oliver and Morest, 1984), neurotransmitter type (Oliver et al., 1994; Saint Marie, 1996), intrinsic firing properties (Sivaramakrishnan and Oliver, 2001), or molecular markers (Ito et al., 2009; Goyer et al., 2019; Schofield and Beebe, 2019; Silveira et al., 2020; Kreeger et al., 2021). At this point, it is unclear whether LDS-induced facilitation is a property of a single neuron type. Both GABAergic and non-GABAergic cells can show LDS-induced afterdischarges *in vivo* (Ono et al., 2016), and PTP can be elicited *in vitro* in sustained-regular, rebound and build-up pauser cells (Wu et al., 2002). Some IC neurons have a non-monotonic response pattern (e.g., they have reduced firing rates at higher stimulus intensities) (Wang et al., 1996; Ono et al., 2017) that could be due to an intrinsic property or a non-monotonic input. It is theoretically possible that a momentary synaptic adaptation or TTS resulting from an LDS might lead to increased firing in non-monotonic neurons sufficiently to induce LSA. However, it may be rare since the afterdischarge response is positively correlated with the intensity level of the LDS.

The mechanism for LDS-induced facilitation could involve the activation of metabotropic glutamate receptors (mGluR). Activation of group 1 and 2 mGluRs increases spontaneous activity in the IC, and group 2 mGluR activation can enhance sound responses (Voytenko and Galazyuk, 2011; Kristaponyte et al., 2021). mGluR are located perisynaptically on the presynaptic or postsynaptic terminal depending on their subtype (Baude et al., 1993; Nusser et al., 1994; Shigemoto et al., 1997; Niswender and Conn, 2010). The perisynaptic localization of the mGluRs necessitates a high amount of glutamate release for their activation (Baude et al., 1993; Nusser et al., 1994). Therefore, if LDS-induced facilitation requires mGluR activation, their perisynaptic localization could explain the dependency of LSA and tone-evoked response facilitation on the LDS response strength and LDS level (Figures 5, 10) and duration (Ono et al., 2016).

Long-duration sound-induced facilitation could be a property of the local circuit in the IC. The IC is known for its extensive local circuitry and the resulting recurrent local feedback. Disk-shaped neurons in the central nucleus, for example, make extensive local collaterals that are confined to the same fibro-dendritic lamina (Oliver et al., 1991; Ito and Oliver, 2014). These local collaterals provide for extensive polysynaptic activity within the IC (Sivaramakrishnan et al., 2013) and could play a major role in the formation of new auditory coding properties in the IC (Atencio et al., 2016; Ito and Malmierca, 2018). The data here is consistent with the idea that the LDS-induced facilitation may activate multiple, distant neurons within the same fibro-dendritic laminae; however, with LDS levels of 73 dB SPL many laminae are likely stimulated.

### Population Coding in the Inferior Colliculus

Plasticity is an important tool that allows the brain to adapt to changes in the environment. These changes may be on the level of a single synapse or a whole circuit. Our data suggests that a LDS is able to induce a form of short-term plasticity in the IC. The role of short-term plasticity is thought to lie in its influence on information processing at the synapse (Citri and Malenka, 2008). The LDS may act similarly to a tetanizing stimulus that causes PTP, LTD, and LTP in IC neurons *in vitro*. The LDS acts to potentiate some neurons, while others are suppressed or unaffected. The net effect of the LDS is a shift in the population activity that would, in turn, alter the output of the IC. Thus, the information carried by the neurons with sound-evoked facilitation is increased in the output of the IC, while the influence of the other neurons is diminished. Since the main projections of the IC are to the thalamus and contralateral IC, the forebrain responses after repetitive or continuous acoustic stimuli with long durations may be altered due to the shift in the responses in the ICC population.

How might this short-term plasticity impact hearing? When a longer duration sound is heard, the population activity in IC shifts so that facilitated neurons contribute a larger proportion of the IC output to the forebrain. Facilitation is most likely to occur in neurons that are the most strongly driven by the stimulus. Thus, the circuits responding best to the stimulus are strengthened, while other circuits are suppressed by adaptation. This may result in a transient improvement in sensitivity to that long duration sound after it ends. Such increased sensitivity may only last a few minutes as the circuits return to the “normal” balance and output. Thus, this plasticity may result in a transient altered state in the auditory pathway where some acoustic stimuli are favored over others, and it might play a role in some types of auditory learning.

The afterdischarge occurs when there is no sound stimulus; it is after the offset of the sound. In that sense it resembles tinnitus. Noise-induced hearing loss can result in increased spontaneous neural activity throughout the central auditory pathway that is thought to stem from the lack of activity in the cochlea (Kaltenbach and McCaslin, 1996; Wang et al., 2002; Seki and Eggermont, 2003; Herrmann and Butler, 2021). It is possible that the mechanisms responsible for LSA and sound-induced facilitation become pathologically activated after noise-induced hearing loss and continue to produce the never-ending increased level of spontaneous activity associated with tinnitus. Insights gained into the mechanisms of sound-induced facilitation could shed light on the pathological mechanisms in tinnitus.

## Conclusion

Subcortical auditory responses are subject to plasticity, and the exact mechanisms are still being investigated. This study demonstrates a novel type of sound-dependent plasticity in the inferior colliculus where a LDS changes the subsequent tone-evoked responses. This plasticity is not uniform. A subpopulation of neurons shows a facilitation of tone-evoked responses after the long duration sound, while others show no effect or a suppressed response. As the IC directly synapses with the thalamus, these changes in the pattern of sound encoding can affect further upstream auditory processing.

## Data Availability Statement

The raw data supporting the conclusions of this article will be made available by the authors, without undue reservation.

## Ethics Statement

The animal study was reviewed and approved by Animal Care and Use Committee at the University of Connecticut Health Center.

## Author Contributions

AB: study design, data collection, analysis, and writing – first draft. CL: study design, data collection, code writing, and writing – first draft. EF-S: data collection, writing – review and editing. DO: study design, writing – first draft, and funding acquisition. All authors contributed to the article and approved the submitted version.

## Conflict of Interest

The authors declare that the research was conducted in the absence of any commercial or financial relationships that could be construed as a potential conflict of interest.

## Publisher’s Note

All claims expressed in this article are solely those of the authors and do not necessarily represent those of their affiliated organizations, or those of the publisher, the editors and the reviewers. Any product that may be evaluated in this article, or claim that may be made by its manufacturer, is not guaranteed or endorsed by the publisher.

## Abbreviations

LDS: long-duration sound
LSA: long-duration sound evoked afterdischarge.
