# Supplementary figures and images for: Long-Duration Sound-Induced Facilitation Changes Population Activity in the Inferior Colliculus
Source: Front Syst Neurosci. 2022 Jul 7;16:920642. doi: 10.3389/fnsys.2022.920642 (PMC9301083; doi:10.3389/fnsys.2022.920642)

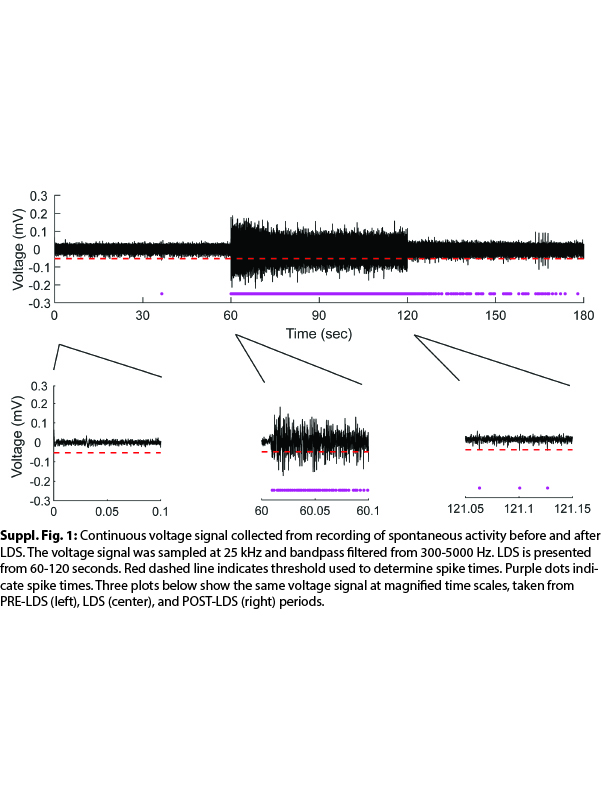

Supplement: Supplementary file 1 [file Image_1.JPEG]

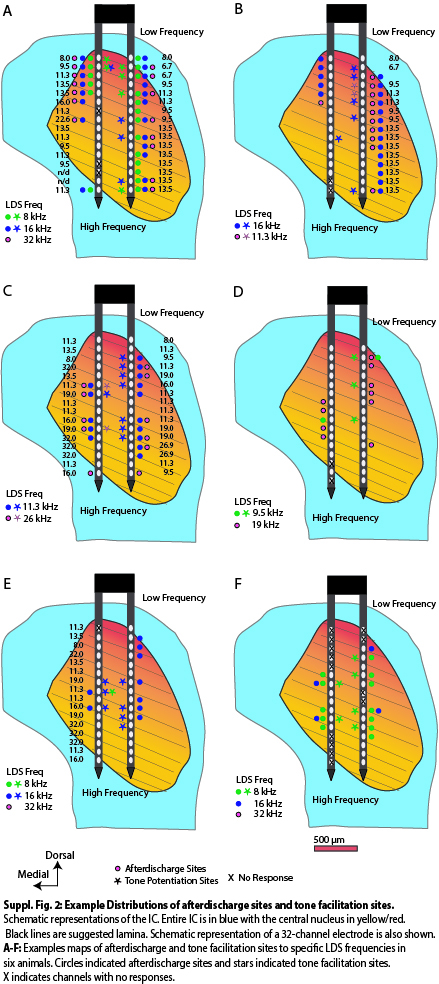

Supplement: Supplementary file 2 [file Image_2.JPEG]

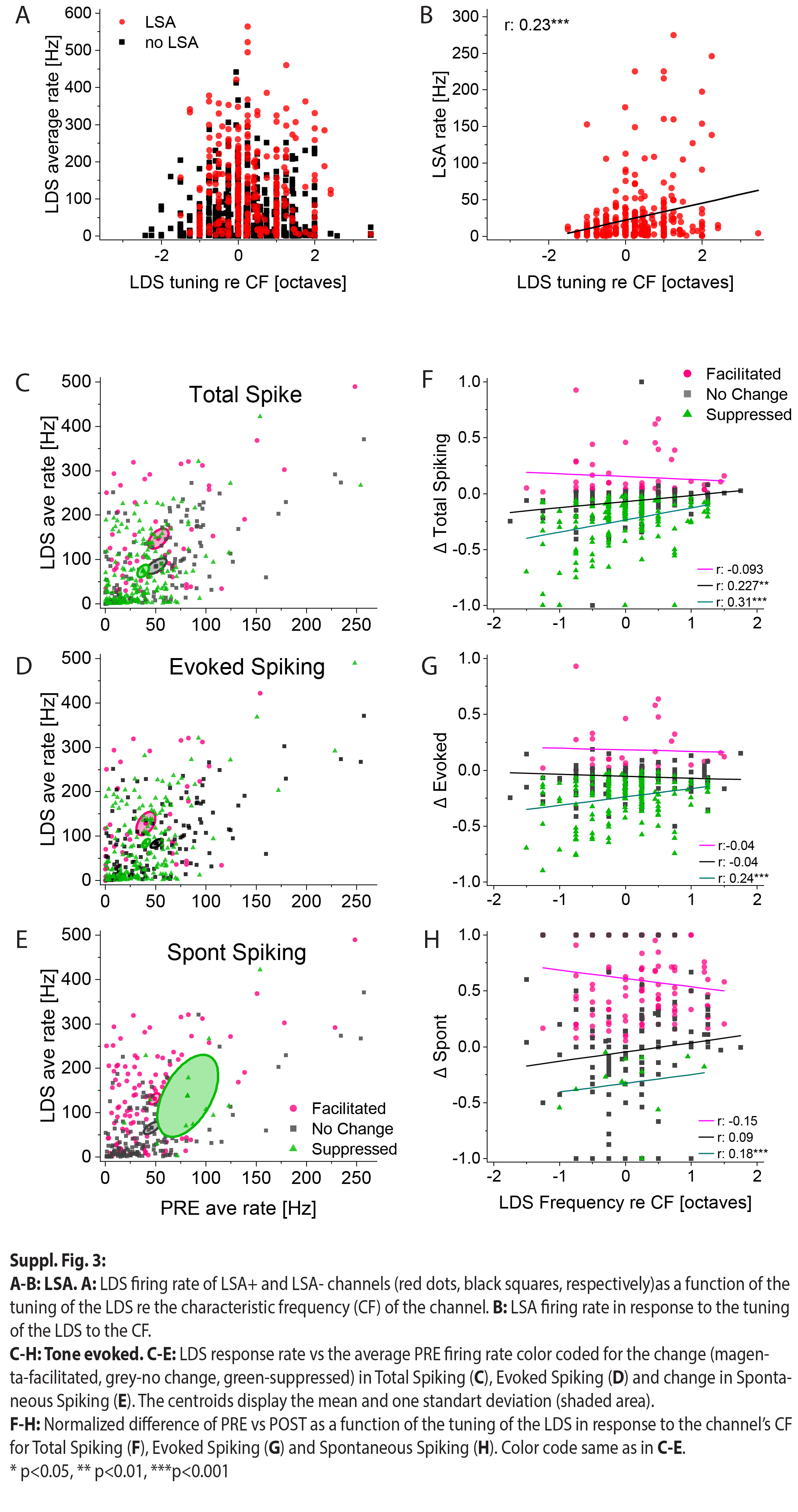

Supplement: Supplementary file 3 [file Image_3.jpg]

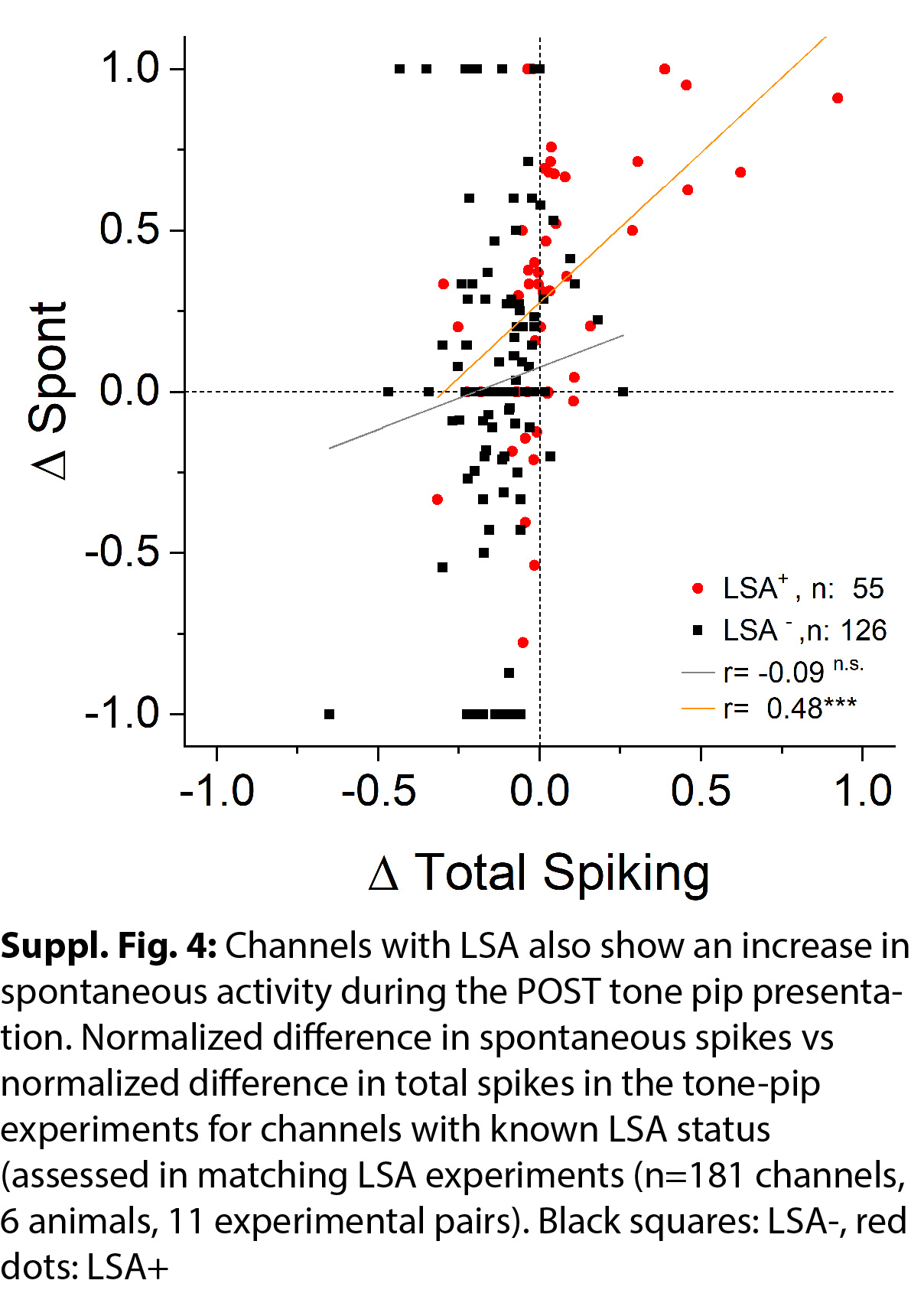

Supplement: Supplementary file 4 [file Image_4.jpg]
